# The Design of a Dynamic Configurable Packet Parser Based on FPGA

**DOI:** 10.3390/mi14081560

**Published:** 2023-08-05

**Authors:** Ying Sun, Zhichuan Guo

**Affiliations:** 1 National Network New Media Engineering Research Center, Institute of Acoustics, Chinese Academy of Sciences, No. 21, North Fourth Ring Road, Haidian District, Beijing 100190, China; suny@dsp.ac.cn; 2 School of Electronic, Electrical and Communication Engineering, University of Chinese Academy of Sciences, No. 19(A), Yuquan Road, Shijingshan District, Beijing 100049, China

**Keywords:** FPGA, packet parser, dynamic configurable, low lantency

## Abstract

To meet the evolving demands of programmable networks and address the limitations of traditional fixed-type protocol parsers, we propose a dynamic and configurable low-latency parser implemented on an FPGA. The architecture consists of three protocol analysis modules and a TCAM-SRAM. Latency is reduced by optimizing the state machine and parallel extraction matching. At the same time, we introduce the chain mapping idea and container concept to formulate the matching and extraction rules of table entries and enhance the extensibility of the parser. Furthermore, our system supports dynamic configuration through SDN control, allowing flexible adaptation to diverse scenarios. Our design has been verified and simulated with a cocotb-based framework. The resulting architecture is implemented on Xilinx Ultrascale+ FPGAs and supports a throughput of more than 80 Gbps, with a maximum latency of only 36 nanoseconds for L4 protocol parsing.

## 1. Introduction

In recent years, programmable networks [1,2,3,4,5] have gained significant attention due to their flexibility and adaptability to meet the evolving needs of modern communication networks. Packet parser plays a crucial role in network communication [6,7], where network nodes extract specified data fields from packets according to protocol specifications for lookup and forwarding operations [8]. Traditional software-based packet parsers suffer from limited processing capabilities and high latency [9]. To enhance the processing capacity of network devices, dedicated hardware implementations such as Application-Specific Integrated Circuits (ASICs) are commonly employed [10]. However, ASIC-based systems have long development cycles and high production costs, limiting their flexibility and scalability. In contrast, Field-Programmable Gate Array (FPGA)-based solutions offer a favorable trade-off between high data rate processing and flexibility [11,12,13], making them an ideal choice for Software-Defined Networking (SDN) [14].

Currently, common FPGA-based solutions adopt a multi-stage pipeline architecture for packet parsing [15,16,17,18,19,20,21], this approach assigns a separate parsing module to each protocol layer or type, resulting in a significant hardware resource overhead. Moreover, it fails to support packet parsing beyond the limit of available parsing modules, thus limiting parsing flexibility. When the parsing graph changes, reconfiguring the FPGA becomes necessary to accommodate new parsing requirements. An instruction-based parser architecture is proposed in [22,23,24]. Similar to pipelined computer processors, instructions need to be read from memory, and then execution units are assigned to perform extraction and reorganization operations according to the instructions. These processes result in high latency. To overcome these challenges, this paper proposes a solution for implementing a dynamic and configurable packet parser on an FPGA. Our proposed approach utilizes a single parsing module in a cyclic structure to effectively reduce the hardware resource overhead introduced by multiple parsing units. By employing parallel extraction and matching techniques, we achieve lower latency while allowing dynamic configuration based on specific requirements. We verify and simulate our design using the cocotb framework. The resulting architecture is implemented on Xilinx Ultrascale+ FPGAs and supports a throughput of more than 80 Gbps, with a maximum latency of only 36 ns for L4 protocol parsing. The primary contributions in this paper are as follows:

- The architecture proposes a novel parallel extraction matching module architecture so that the type of the next layer protocol is matched in parallel while extracting key fields. This reduces the waiting time for the return of the extraction rules.
- A new extraction rule based on offset-container is proposed. Compared with traditional offset-length-based extraction, it reduces hardware overhead and has excellent scalability.
- We use the control plane to send FlowMod messages to realize the dynamic configuration of the parser at runtime. Network engineers avoid FPGA development and use the special API to provide useful configuration data based on dynamic development application needs.

## 2. Related Work

Kozanitis et al. [15] introduced a unique Kangaroo parsing architecture that stores packets in memory and employs on-chip associative memory for speculative lookahead of stored data. However, this approach suffers from high latency, making it incompatible with today’s switches.

Attig and Brebner [16] proposed a 400 Gbps programmable parser for Xilinx Virtex-7 FPGAs. Their method includes a domain-specific language for describing packet parsers, modular and pipelined hardware architecture, and a parser compiler. While the deep pipeline in this architecture allows for high throughput, it faces challenges in terms of latency and resource utilization.

Gibb et al. [25] presented a detailed design of fixed and programmable high-speed packet parsers. The packet parser design in the Register Management Table (RMT) is relatively simple and relies on associative memory. Both works assume ASIC as the target implementation platform and thus do not demonstrate FPGA-based results.

Benácek et al. [17] proposed an automated high-speed P4 to VHDL packet parser generator targeting FPGA platforms. The hardware architecture of the packet parser consists of a set of configurable parsing engines [26] organized in a pipelined manner. The generated parser achieves 100 Gbps for a set of fairly complex headers. However, the results show that compared to the hand-written VHDL implementation, there is a greater overhead in terms of latency and resource consumption.

Cabal et al. [18] presented a method of placing multiple packets on a data bus to supply data to a parallel deep-pipeline parser, which can scale to Tbps throughput on Xilinx UltraScale+ FPGAs.However, high-bandwidth data transfer requires demanding transmission media, making the implementation complex.

HyperParser [27] proposed a high-performance parser architecture targeting next-generation programmable switches and FPGA-based SmartNICs. Its butterfly network is optimized for packet parsing performance, logic resource utilization, and device power. While this solution offers significant improvements in performance and supports both ASIC and FPGA deployments, protocol updates require at least several tens of seconds for loading.

Wang et al. [28] proposed an ICN dynamically extensible protocol parser based on the FPGA platform. They introduced the extended protocol descriptor and multi-queue protocol management mechanism, enabling dynamic updates and efficient parsing of customized ICN protocol rules. The parser supports the flexible expansion of new protocol parsing rules at the end of the protocol parsing tree. However, it lacks support for inserting new protocols in the middle of the original parsing process or even at the root position.

Li et al. [29] proposed a programmable packet-level parallel parsing architecture for FPGA-based switches. They utilized packet-level parallelism in the parsing pipeline bottleneck to compensate for the FPGA’s low clock frequency and reduce resource consumption without replicating multiple parsing blocks. Compared to non-parallel strategies, the parsing performance improved by a factor of 10. However, due to combining different header types in a single entry during predictive parsing, the number of entries in the lookup exhibited exponential growth.

Refs. [22,23,24] proposed packet parsers designed for instruction-based architectures, which are similar to pipeline computer processors where packet processing is performed based on commands specified by instructions. The main advantages of instruction-based parsers lie in their flexibility and configurability. However, instruction-based packet parsers can result in higher processing latency. Ref. [24] proposed instruction reuse schemes and storage structures to effectively improve header extraction latency.

In summary, the related work in this field has explored various methods for high-speed packet parsing. However, existing works still face challenges such as high latency, resource consumption, protocol update time, or limited compatibility with FPGA-based implementations. Therefore, more efficient designs are needed that strike a balance between latency and resource utilization. Compared to the works in [15,16,17,18,19,20], our single-block recursive layout results in lower resource consumption. By employing a protocol-agnostic generic architecture we eliminate the need for generating multi-level structured parsing units. In comparison to [25], we reduce processing latency caused by protocol dependencies through parallel extraction and matching. In addition, we provide a set of FlowMod interfaces that enable users to flexibly configure the parsing protocols.

## 3. Model Description

### 3.1. System Structure

**Figure 1:**
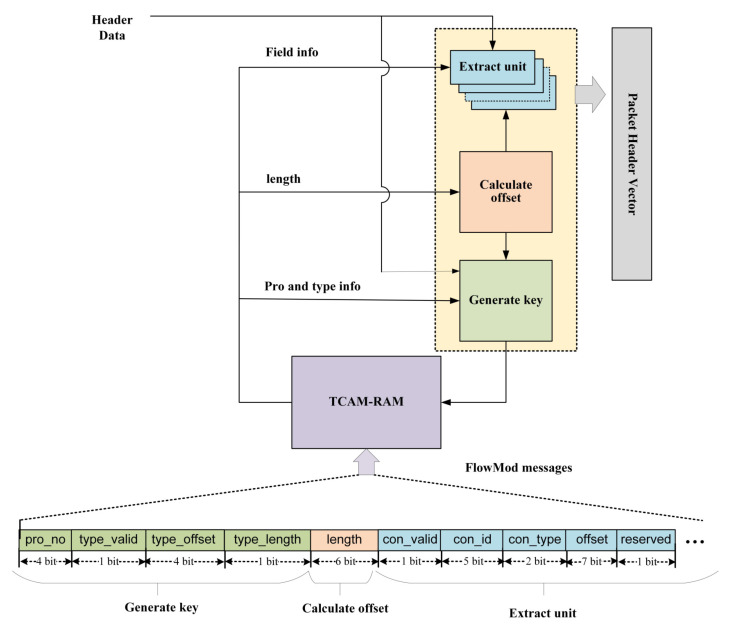
The diagram of parser.

The Dynamic Configurable Packet Parser (DCPP) implemented in this paper, based on FPGA, is designed to extract specified data fields from packets received from Ethernet and combine them into a packet header vector (PHV) for subsequent lookup and forwarding processing. The architecture, as shown in Figure 1, primarily consists of three protocol analysis modules and a TCAM-SRAM. The protocol analysis modules are responsible for processing and determining the necessary actions for the input packets. By segregating different parsing functionalities into separate modules, this architecture enhances the parallelism and efficiency of the parsing process. In addition, the utilization of TCAM-SRAM as a high-speed lookup table enables fast packet matching and filtering, thereby accelerating the parsing speed and reducing processing time.

Specifically, the key field extraction and protocol type recognition module of the parser receives packet fragments from Ethernet. After extracting and matching the Ethernet protocol type, the information stored in Random Access Memory (RAM) is processed in parallel by three protocol-agnostic analysis modules: extract unit, calculate offset, and generate key. This parallel processing significantly reduces the waiting time of each module. The protocol parsing is an iterative process that continues until the table entry explicitly indicates that the module has reached a leaf node. Eventually, the extracted field domains are combined into a high-width packet header vector and sent to the subsequent matching and lookup stage.

In addition, the bottom arrow represents users providing matching fields and extraction rules for processing parse graph messages through the upper-level application programming interface. These rules are stored in TCAM and RAM by issuing FlowMod messages. This approach allows for the customization of arbitrary protocols.

### 3.2. Optimized State Machine Design

**Figure 2:**
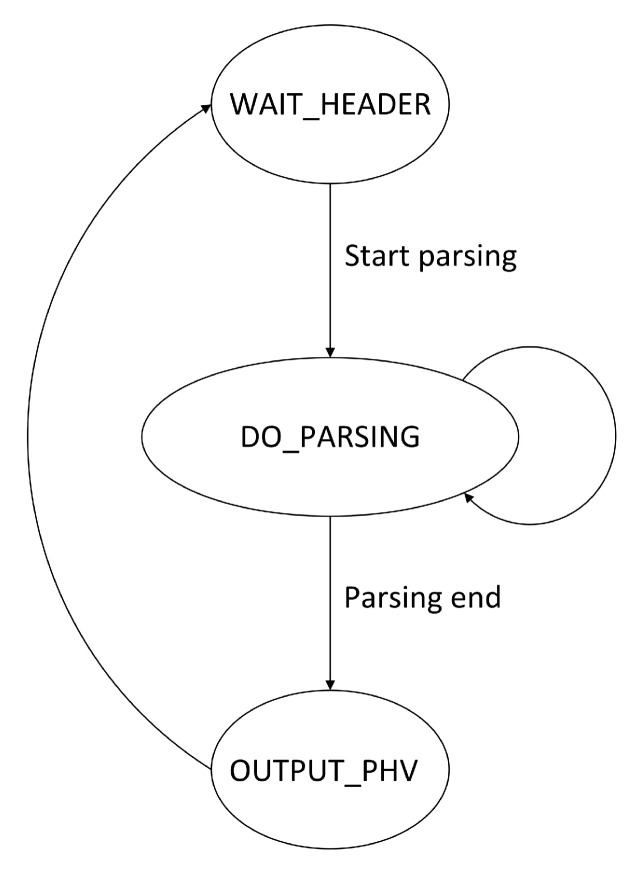
State machine diagram.

In the hardware code implementation, we have addressed the drawbacks of previous approaches that rely on state machine jumps for parsing different protocol layers. State machine jumps refer to the transitions between different state machines to process various protocol layers. When the current state machine finishes processing one protocol layer, it switches to another state machine to process the next layer. These jumps often lead to high latency and limit the number of protocol layers that can be processed by the state machines. To overcome these issues, this paper proposes an optimization in the design of the state machine. The design integrates the parsing logic of different protocols into a single-state machine. The state machine can determine the corresponding state transitions and processing operations based on the currently parsed protocol type. This approach enables the parsing of multiple protocols within a single state machine, eliminating the latency caused by state machine jumps and reducing the number and complexity of state machines. The specific parsing process is illustrated in Figure 2.

Step 1: The protocol parsing begins when the header is received. For a given network, the initial header type is typically known to the parser (e.g., Ethernet). The key field module extracts the header type value based on the packet’s header offset and sends the type field information to the TCAM.

Step 2: The initial header processing leads to the entry of the protocol parsing state machine. Within this state machine, the matching and processing operations are performed iteratively. The state machine receives RAM information and extracts the next-layer protocol type, calculates the next-layer protocol’s starting offset based on length, and extracts the key fields based on the received information. If the RAM information is valid, the three extraction engines work in parallel until the RAM returns information indicating that the state machine has reached the last protocol layer.

Step 3: After the extraction is completed, the state machine enters the termination state to output the valid packet header vector and returns to the initial state.

### 3.3. Parallel Extraction and Matching

The TCAM-based packet parsing process consists of six steps: (1) protocol type identification; (2) TCAM matching; (3) RAM retrieval of protocol extraction rules; (4) extraction of key fields based on the rules; (5) calculation of protocol length based on the extracted length fields; (6) assemble the extracted key fields into a packet header vector.

Firstly, the protocol type identification step locates the protocol header and extracts the current protocol type field, which serves as a crucial parameter for the subsequent TCAM Matching. Secondly, the TCAM matching step matches the next layer’s protocol type based on the extracted type field, leading to the retrieval of essential information from RAM in the third step. The RAM retrieval of protocol extraction rules provides critical data, including length, key field positions, and corresponding extraction rules. Leveraging this information, the extraction of key fields, the fourth step, accurately captures fields from the packet header based on the position information. Subsequently, the calculation of protocol length, the fifth step, determines the starting offset of the next layer by evaluating the length of the current layer’s protocol. Finally, the assembly of extracted key fields, the sixth step, consolidates the extracted information into a packet header vector for further processing.

The main challenge of this parsing model is the low processing performance due to the interdependencies of protocol parsing. The protocol type identification module needs to wait for the TCAM matching operation to combine the extracted key fields into a packet header vector. After the Ethernet protocol is processed, the IPv6 protocol is parsed. Processing one packet requires completing these six steps before parsing the next packet.

Typically, multiple fields need to be extracted for a protocol layer, while the extraction module can only extract one field per clock cycle. The protocol type identification submodule, TCAM submodule, and RAM submodule can receive packets within each clock cycle, but the field extraction module must wait for the previous field extraction to complete before processing the next field. Similar to a congested node in a network, the processing delay for all subsequent packets increases, and packet loss may even occur. As the number of fields to be extracted increases at each layer, the bottleneck effect of this module on performance becomes more prominent.

**Figure 3:**
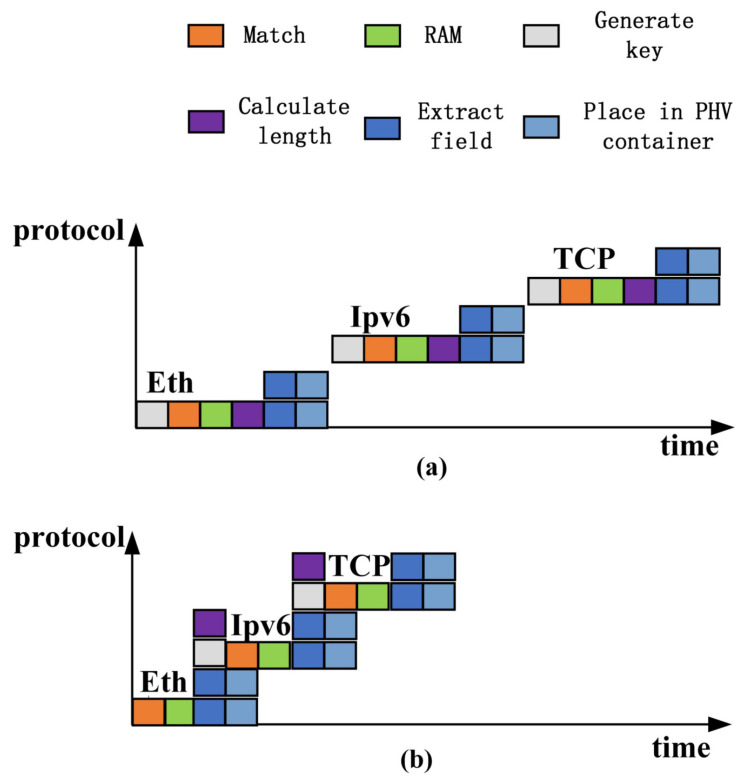
Delay comparison diagram. (**a**) Serial extraction matching delay diagram. (**b**) Parallel extraction matching delay diagram.

Within the same protocol layer, the extraction of key fields can be performed in parallel [25]. To reduce the time overhead of field extraction in the parsing process, we have integrated multiple field extraction units that support simultaneous extraction of fields within a single cycle, as shown in Figure 3a.

Based on our analysis of the dependency relationships in protocol processing, we observed the potential for parallel execution between certain critical operations, particularly in identifying the next layer’s protocol type and calculating the length-based starting offset, as well as in extracting fields and assembling them into a packet header vector output. This parallel execution can significantly reduce the parsing delay and improve system efficiency.

Upon further observation, we noticed that it is feasible to perform identification and matching operations immediately after extracting the next layer’s protocol type, without waiting for field extraction and PHV assembly. This parallel extraction scheme allows for time savings while maintaining data consistency. By doing so, we can begin to match the protocol types of the next layer while extracting fields of this layer, thereby maximizing the utilization of system resources and improving parsing efficiency.

Through the illustrated improvement, we can clearly demonstrate the advantages of this parallel extraction. The modified Figure 3b shows that the wait time between protocol layers has been reduced by half compared to the original Figure 3a, resulting in a doubling of the overall parsing efficiency.

Based on the above observations and analysis, we have optimized the dependency relationships in the protocol processing and implemented a parallel extraction scheme. This scheme reduces waiting time and enables parallel execution of critical operations, resulting in a significant reduction in parsing delay and improvement in overall system performance and efficiency.

### 3.4. Extract Crossbar

In our method, the offset calculation is effectively implemented using an adder. In addition, two key modules, namely the generate key module and the extract module, play essential roles in extracting the required protocol fields and generating the packet header vector. Both the extract module and generate key module are responsible for extracting the required protocol fields from anywhere in the header fragment. Thus, our focus is on the specific analysis of the crossbar. The crossbar operates by establishing direct connections between specific inputs and outputs as needed. When data need to be transferred from a particular input to a specific output, the crossbar creates a temporary pathway by connecting the corresponding switching element. This direct link ensures efficient and non-blocking data transmission between the selected input and output.

In this article, we employ a zero-extended crossbar [30], which is capable of reading data of different widths from its input and output data in a larger fixed-width format, with zero-padding unused bits. By utilizing two-stage crossbar switches with zero scalability, our approach leads to a remarkable 52.7% reduction in area and a significant 46.7% decrease in power consumption compared to conventional bit-level interconnect schemes [30]. The advantage of this reduction is derived from the ability of the crossbar switch to handle varying data lengths without requiring additional resources for expansion, unlike bit-level interconnect schemes that tend to be more complex and resource intensive.

**Figure 4:**
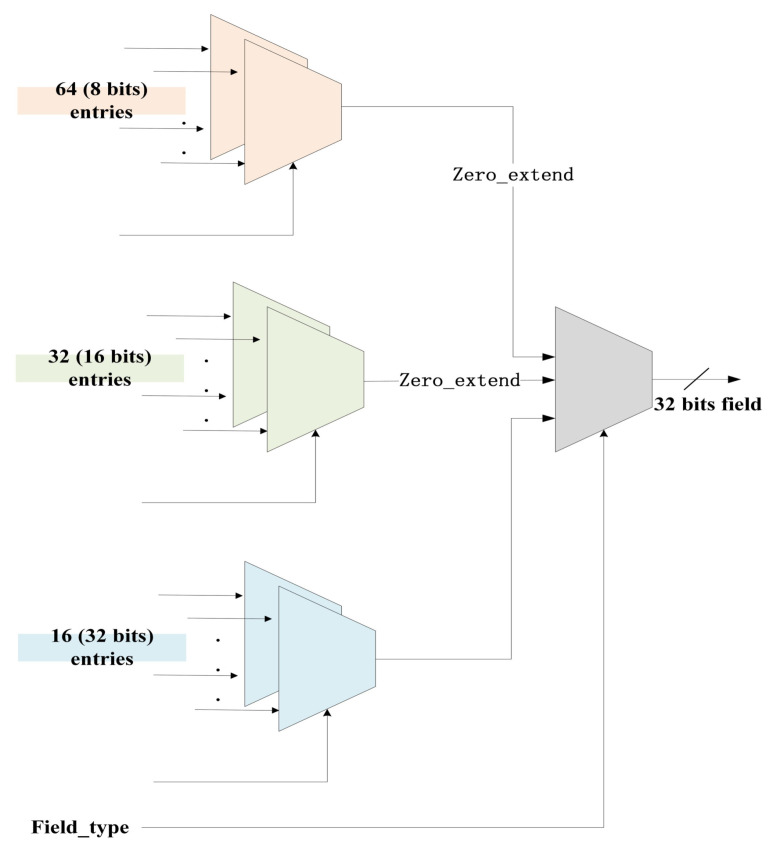
The diagram of crossbar in the extract module.

Figure 4 illustrates the crossbar configuration employed in our extract module when the input data width is 1024 bits. In this setup, the crossbar consists of a total of 112 inputs, comprising 64 inputs with 8-bit data (8-bit entries), 32 inputs with 16-bit data (16-bit entries), and 16 inputs with 32-bit data (32-bit entries). Since PHV has a maximum bit width entry of 32 bits, our extract module employs a crossbar with an output bit width of 32 bits. This specialized crossbar is designed to handle various input data widths efficiently. It can accept any 32-bit entry directly and also accommodate 16-bit and 8-bit entries with zero extension to 32 bits. For example, when the crossbar reads 8-bit data from one of its inputs, it will automatically output that data in a 32-bit format with 24 bits padded with zeros. Similarly, when it reads 16-bit data from another input, it will output that data in a 32-bit format with 16 bits padded with zeros. This zero-padding ensures that all data in the crossbar have a consistent output width of 32 bits.

**Figure 5:**
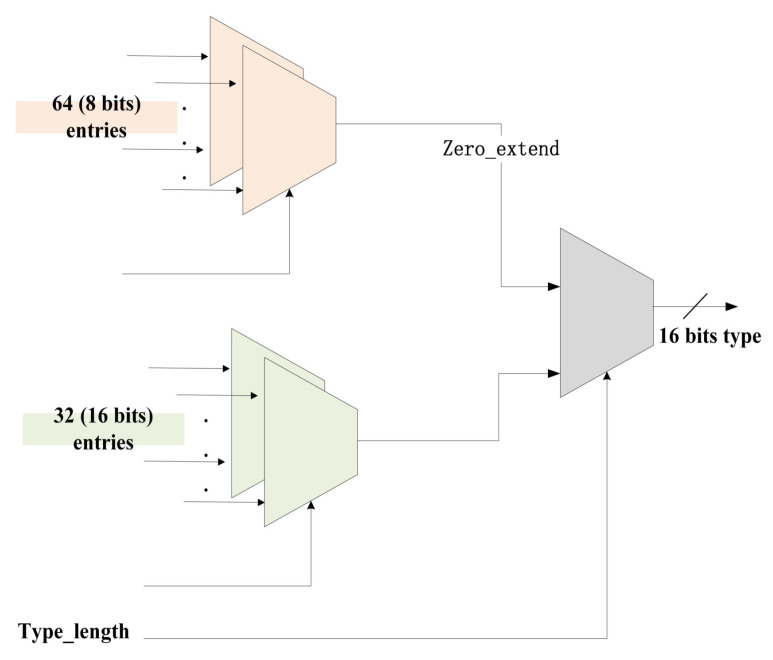
The diagram of crossbar in the generate key module.

For generating the key module, we used two protocol types with 8-bit and 16-bit, which support the judgment of a common base protocol. So we take a crossbar with an output bit width of 16 bits, accepting any 16-bit entry or any 8-bit entry with zero extension to 16 bits, as shown in Figure 5.

### 3.5. TCAM-SRAM Table Structure

This study presents the design of a configurable packet parser aimed at constructing a protocol parsing flow graph through matching and extraction. When a new protocol appears, we do not need to modify the Verilog code, just adding extraction entries in the table can support the parsing of the new protocol. To achieve this goal, we map the parsing flow graph to TCAM matching using a chain structure.

**Figure 6:**
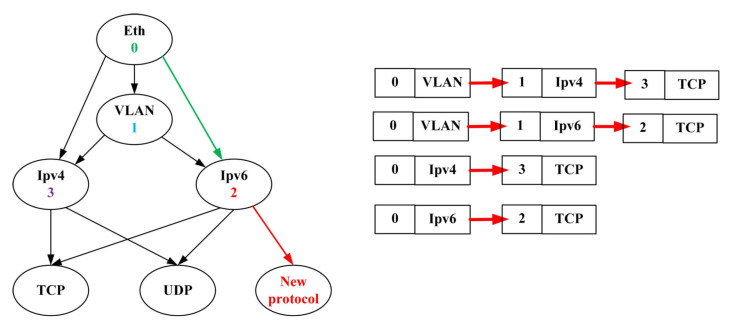
Ethernet protocol parsing tree and chain mapping.

The encapsulation of the headers of each protocol layer is based on the layers through which the data passes at the sender side. Therefore, when parsing an Ethernet packet, it needs to be parsed in a specific order. If we consider the protocol of each layer in the model as a vertex in the parsing graph and the transition to the next layer protocol header as an edge in the parsing graph, we can construct a parsing graph for the packet parser in the network device. Taking the common protocols included in an Ethernet packet as an example, we construct an Ethernet protocol parsing graph as shown in Figure 6.

**Figure 7:**
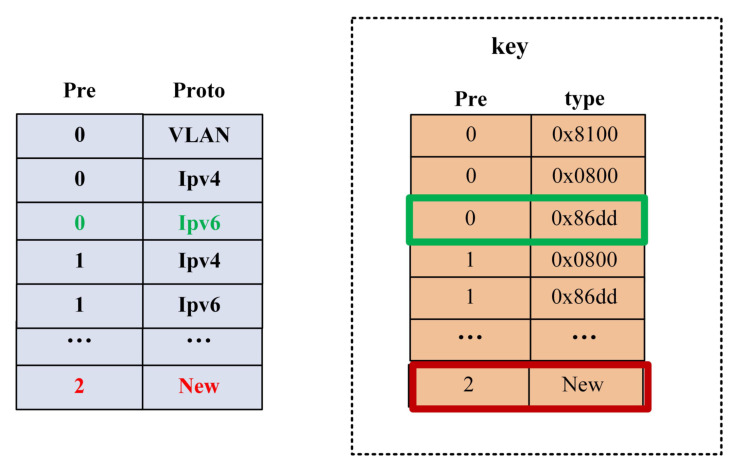
TCAM matching entries.

During the packet parsing process, the parsing of protocols is interdependent. In the parsing graph, the head node and the information pointing to the tail node uniquely determine the tail node. Each edge represents a transition process, and we focus on describing the transitions between different protocols based on edges. Firstly, each edge is sequentially labeled with the corresponding number of its head node. In the parsing process, each protocol has a unique identifier. We can use the head node and the agreement of the current node type to represent each edge, which serves as the matching field for TCAM. In this way, all the edges are stored in the TCAM-SRAM, where the number of edges in the parsing graph equals the number of TCAM entries, as shown in Figure 7. When adding a new protocol, it can be merged into the parsing graph by linking it to the head node of existing edges, and only new rules need to be added in the TCAM-SRAM entries to support the extraction of the new protocol.

**Figure 8:**
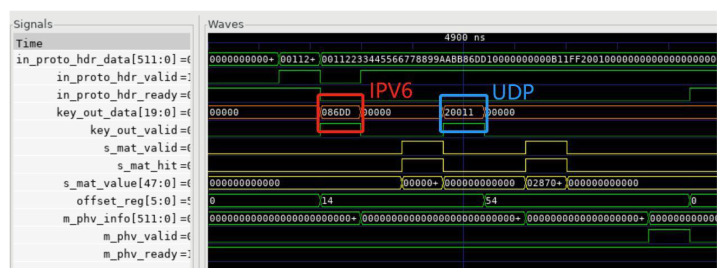
TCAM matching simulation diagram.

As an example, we simulated the matching process of the parsing flow graph Eth-IPV6-UDP using cocotb, as shown in Figure 8. The highlighted data are extracted by the “generate_key” module and subsequently used for matching in the TCAM. The “generate_key” module is responsible for extracting key fields from the header fragment to create a key used for protocol type identification. The first highlighted number represents the key extracted when transitioning from the Ethernet protocol to IPv6. In this case, the Ethernet protocol is denoted by sequence number 0, and the IPv6 protocol type is represented by the hexadecimal value 0x86dd. The second highlighted datapoint represents the key extracted when transitioning from IPv6 to UDP. The IPv6 protocol is indicated by sequence number 2, and the UDP protocol type is represented by the hexadecimal value 0x11.

In the work [17], the data extractor adopts extraction rules based on offset and length. By inputting the offset, the position of the current protocol field to be parsed in the packet header is determined, while the length is used to determine the number of bytes to extract from the packet data. Theoretically, when the bit width of the offset and length is large enough, the data extractor can extract data of any length from any byte position in the group bus data word. However, the implementation of multiplexers inside the data extractor introduces significant resource overhead. In addition, there are limitations to the bit width of the length. When the key field of a new protocol exceeds the defined length bit width, it faces issues with extraction.

**Figure 9:**
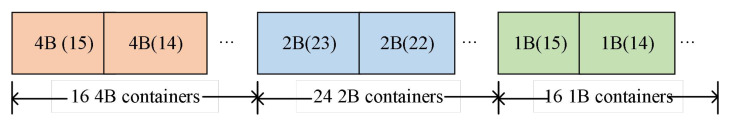
PHV container arrangement diagram.

**Figure 10:**
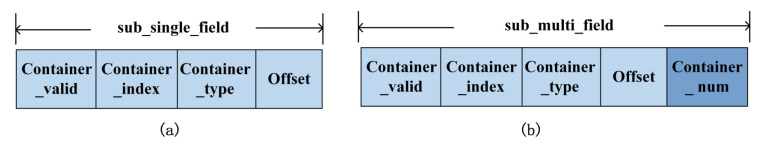
Extraction rules based on offset and container. (**a**) Single domain extraction. (**b**) Multi-domain extraction.

According to the description in [31], the parser used in Tofino adopts an output format based on containers, which means the extracted header fields are placed in containers with widths of 8, 16, and 32 bits. These containers form a vector of fields that can be processed in parallel. In our parser, we also adopt a similar output format. Let us consider an example where the packet header vector has a bit width of 1024 bits. In this case, the PHV is composed of 16 containers of 1 byte (8 bits each), 24 containers of 2 bytes (16 bits each), and 16 containers of 4 bytes (32 bits each). The arrangement of the containers is depicted in Figure 9 below:

This organization allows for efficient parallel processing of header fields, providing flexibility and scalability to handle diverse data widths encountered in the packet headers. Therefore, we can replace the term “length” in the above discussion with the type of container. As shown in Figure 10a, there are three container types in our implementation. This design allows us to represent the widths of different containers using only 2 bits for the “Container_type”, reducing the overhead of multiplexers. In addition, the “Container_index” is used to determine the position of the field in the packet header vector. With the offset-container-based extraction rules shown in Figure 10a, we can not only determine the position of the field in the input packet header but also its position in the output packet header vector. This design enables more efficient extraction and processing of header fields and provides convenience for parallel processing.

Meanwhile, when the length of the field exceeds the maximum width of the container, we can achieve the extraction of arbitrary length fields by overlaying multiple “sub_single_fields”, thus achieving stronger scalability. As shown in Figure 10b, for this purpose, we introduce the concept of “sub_multi_field”. Here, “Container_num” represents the number of containers occupied by the field in the PHV. This improvement allows us to handle fields with lengths exceeding the width of a single container and adapt flexibly to the extraction requirements of different-length fields.

**Figure 11:**
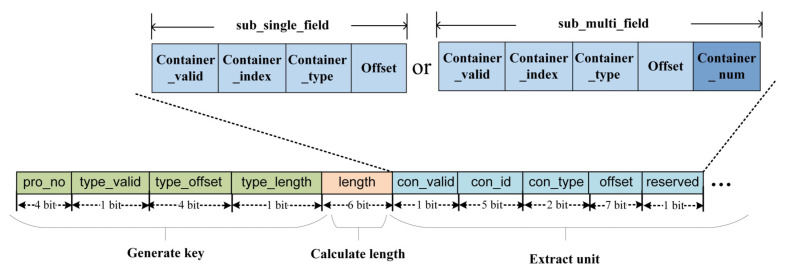
RAM storage information.

TCAM indexes the RAM addresses based on different matching results. The RAM stores processing information for three modules: generate key, calculate offset, and extract unit, as shown in Figure 11. In this study, the parser extracts header fields and places them in three different-sized buffers, namely 8-bit, 16-bit, and 32-bit entries.

For the information format used by the generate key module, the green part represents the following contents: pro_no represents the protocol number; type_valid indicates whether the next-layer protocol type exists. When this value is 0, it means that the current entry has been parsed to the last layer, the loop can be terminated, and the PHV can be outputted. type_offset indicates the offset position of the protocol type in the current protocol. To simplify storage resources, type_length is represented by 1 bit, indicating the number of bits occupied by the protocol type, i.e., 8 bits or 16 bits.

For the information used by the calculate offset module, the orange part represents the following contents: using the total length of this protocol, length, to calculate the starting offset of the next-layer protocol.

For the information used by the extract unit module, the blue part represents the following contents: con_valid indicates whether the extraction rule for this key field is valid; con_id represents the index value of this key field in the PHV different entries; con_type represents which entry of the three types of placeholders the key field is placed in the PHV. This not only determines the length of the key field to be extracted but also determines the position of the key field in the PHV. In addition, offset is used to determine the position of the key field in the header grouping.

### 3.6. FlowMod Configuration Interface

**Figure 12:**
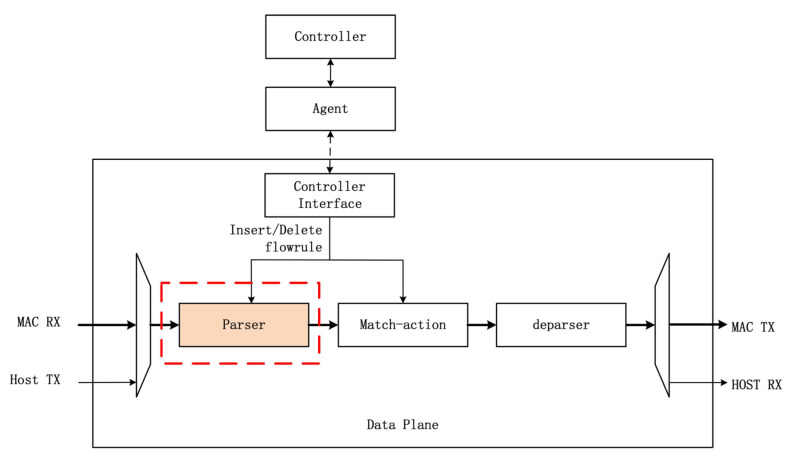
System architecture.

The SDN mode separates the data plane and control plane of network devices to achieve flexibility [32,33]. This article adopts the configuration shown in Figure 12. The control plane runs on the software program agent of the host, responsible for parsing the upper-level configuration of the parser. The data plane runs on the hardware of the FPGA board, and the controller interface module is used to parse FlowMod messages for configuring the TCAM-SRAM of the parser.

**Figure 13:**
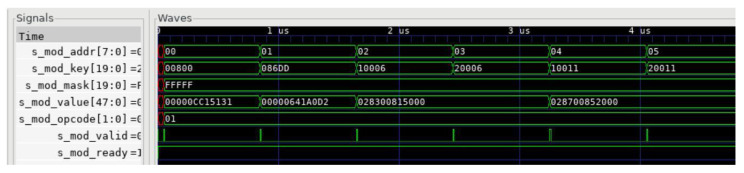
FlowMod configuration interface diagram.

Our parser provides a set of external interfaces for FlowMod messages, and we have simulated the FlowMod configuration interface using cocotb, as shown in Figure 13. In the figure, “s mod addr” represents the storage address, “s mod key” and “s mod mask”, respectively, represent the matching fields of the TCAM and their masks, “s mod value” represents the protocol information and extraction rules stored in the RAM, “s mod opcode” represents the operations of adding, deleting, and querying table entries, and “s mod valid” and “s mod ready” are used as handshake signals for the FlowMod message.

As a result, network engineers avoid FPGA development and simply use dedicated API interfaces that provide useful data based on dynamically evolving application requirements and mask the implementation details of the underlying hardware code. This parser is configurable due to the parameters sent by the software application. This allows dynamic runtime configuration of the protocol being processed without shutting down the controller.

To facilitate the FPGA configuration process, our parser employs a specialized interface for handling FlowMod messages, which are essential for configuring the parser’s TCAM-RAM. These messages are encapsulated by the software application agent and transmitted to a virtual network card. The network card driver further transforms the messages into AXIS control flows using DMA. The controller interface within the FPGA decodes the FlowMod messages to configure the parser accordingly.

Once the FPGA is configured, we do not need a dedicated proprietary tool to configure the parser. The proposed FlowMod configuration ensures efficient software integration and easy adoption by the end user.

## 4. Implementation

**Figure 14:**
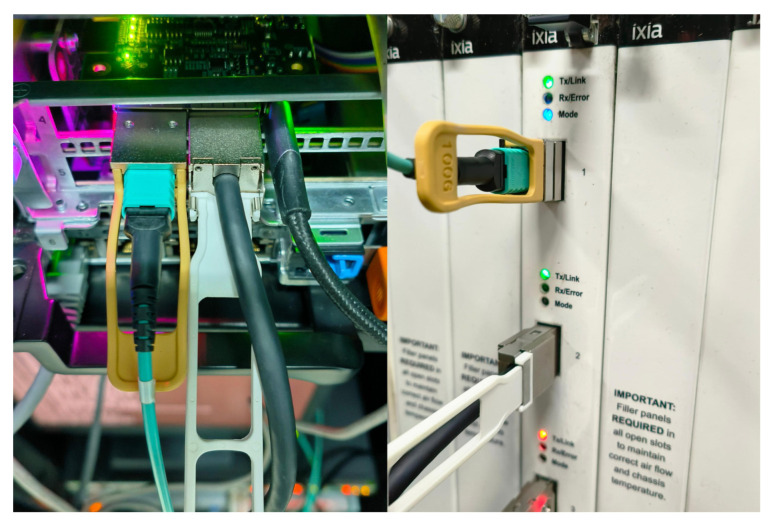
Hardware testbed.

Based on the design and implementation of the DCPP parser proposed above, we deployed the Xilinx Zynq UltraScale+ XCZU19EG-FFVC1760-2-E FPGA on a Dell R740 commodity server. The parser design is described using Verilog HDL and synthesized using Xilinx Vivado 2020.2 for simulation. Subsequently, we downloaded the generated bit files to the FPGA device using Vivado. To generate test data streams, we utilized the IXIA high-speed network traffic generation tool, and the hardware testbed is shown in Figure 14. By combining theoretical analysis and experimental data of DCPP, we evaluated its resources and performance.

### 4.1. Hardware Resource Overhead

The storage overhead in the configurable parser hardware architecture mainly involves the following components: the crossbar for field extraction, TCAM for storing matching fields, and RAM for storing protocol information and extraction rules.

1. Each Ultrascale architecture CLB contains eight 6-input LUTs (LUT6). One LUT6 can be used to implement a 4:1 MUX. By combining adjacent LUT6s, an 8:1 MUX can be achieved. In one CLB, all the LUT6s can implement a multiplexer with a maximum width of 32:1. N LUT6s can be paralleled to implement a 4N:1 multiplexer. For instance, when processing a header fragment with an input width of *I*${}_{B}$ bits, to implement an *I*${}_{B}$:1 multiplexer, we require *I*${}_{B}$/4 LUT6s. In this case, if we want to implement a 64:1 multiplexer (*I*${}_{B}$ = 64), we would need 64/4 = 16 LUT6s. Our calculation is based on using the minimum number of LUT6s. Let *N* represent the number of extract units, *B*${}_{1}$ represent the output width of the extract unit, and *B*${}_{2}$ represent the output width of the generate key module. The total overhead of the extraction crossbar can be represented as:
  (1) $$C_{1}=\left(I_{B}/4\right)\times \left(N\times B_{1}+B_{2}\right).$$
2. The second part is the TCAM-SRAM used for storing matching fields and information. TCAM is used for matching the protocol labels with the protocol type fields. The storage requirement depends on the number of edges in the parsing graph (*E*), the storage width of TCAM (*B*${}_{3}$), and the storage width of RAM (*B*${}_{4}$). The required storage space can be represented as:
  (2) $$C_{2}=E\times \left(B_{3}+B_{4}\right).$$

The overall resource consumption is positively correlated with the protocol width of table entries and the input protocol width of the crossbar. Aggregating the protocol edges through parsing graph fusion can reduce the number of protocol edges, further reducing the number of table entries, and reducing the input protocol width of the crossbar can also lower resource consumption.

To mitigate the impact of different devices on hardware resource usage, we use slice logic (number of LUTs and registers used) as a metric for resource utilization. These devices have the highest utilization in most FPGA designs. The proposed parser was implemented on the aforementioned FPGA and compared with previous studies in terms of resources. Study [28] incurs significant resource overhead due to the introduction of a multi-queue management mechanism, even at the lowest queue depth. Similarly, study [29,34] implements the programmable parsing structure using a single-block recycling approach. From Table 1, it can be seen that by adopting the proposed solution in this paper, the usage of LUTs is reduced by 5.4%, the resource requirement for registers is only 47.3% of that of a non-parallel parser [29], and the overall resource consumption is reduced by 64%. The significant reduction in storage area is attributed to the reduced number of bit selections by the zero-extension crossbar and the reduced width in the proposed storage structure.

### 4.2. Performance Analysis

**Figure 15:**
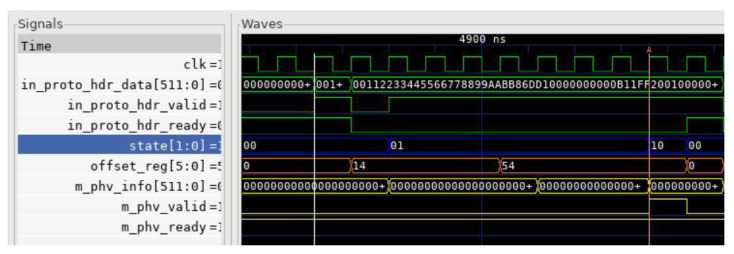
Analysis of the simulation diagram.

Based on the waveform obtained through the cocotb simulation tool, as shown in Figure 15, it can be observed that a total of nine clock cycles were required from the start of parsing to the completion of parsing, resulting in the output of PHV. Table 2 compares the proposed parser design with several previous designs [14,17,28].

**Figure 16:**
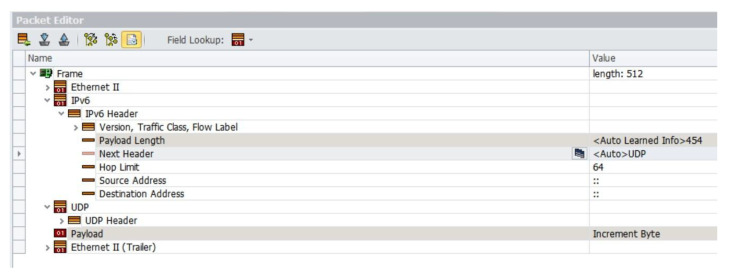
IXIA packet editing page.

The IXIA testers used a custom frame structure to generate traffic, as shown in Figure 16, to set up an Ethernet-IPV6-UDP-based parsing path and analyze the latency. The latency estimation is based on two metrics: clock frequency and the number of clock cycles, in order to standardize the operating frequency and technological differences between designs.

As shown in Table 2, our work, denoted as “Our work${}_{1}$” was implemented on the Xilinx Ultrascale+ FPGA series platform. Despite [14,28] using more advanced FPGA devices than ours, our results exhibit a significant reduction in processing latency. To ensure a fair comparison with [17], we recompiled our system on the Xilinx Virtex-7 XCVH580T FPGA platform used in [17], denoted as “Our work${}_{2}$” in the table. The clock frequency of this method can reach 200 MHz on this platform, and the delay is 45 ns, which is lower than the results reported in [17]. This is because the proposed parallel extraction scheme enables early matching of the protocol types, thereby reducing the waiting time for subsequent modules. Therefore, for scenarios with higher latency requirements, such as live streaming and data centers, this parser has an obvious advantage.

**Figure 17:**
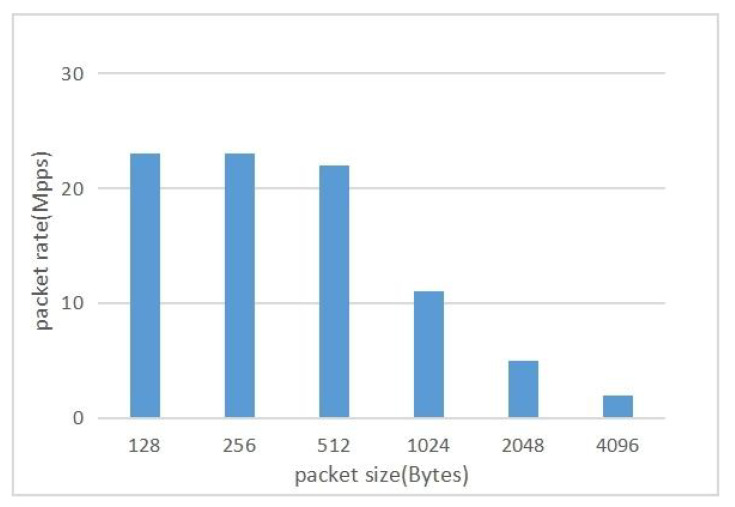
Throughput under different packet sizes.

In our test, we configured the IXIA device to send packets at a rate of 100 Gbps. The performance of the parser was evaluated by adjusting the packet sizes, as shown in Figure 17. The research results indicate that as the packet size decreases, the throughput of the parser exhibits a decreasing trend. This is because smaller packets require the same amount of time for parsing operations, and the module latency has a greater impact relative to the packet length, resulting in increased processing overhead and decreased throughput. Considering the typical size of normal packets in the network, experimental results demonstrate that the parser can efficiently handle packets of 256 bytes or larger, achieving a high throughput of over 80 Gbps. This is significant for networks and communication systems that require the processing of large volumes of data transmission.

In specific application scenarios such as data centers and enterprise networks, common protocols such as Ethernet, IPv4, IPv6, TCP, UDP, etc., have fixed formats, and they do not require high flexibility. In practical applications, these common protocols can be encoded in a fixed manner and processed using single-cycle parsing. This approach reduces the complexity and latency of parsing.

When new protocols need to be added, their protocol information can be stored in TCAM-SRAM table entries for parsing. This approach further reduces resource overhead and latency because only the new protocols require additional storage space, while the parsing of common protocols can be performed using fixed encoding in a single cycle without requiring additional resources.

## 5. Conclusions

Based on the FPGA platform, we propose a dynamic and configurable low-latency parsing design and implementation solution for packet processing in high-speed and low-latency networks. In this solution, we introduce a parallel extraction architecture engine that effectively utilizes early matching of protocol types to reduce waiting time. By using the chain mapping idea and the container concept, we design flexible and efficient matching and extraction rules that enable the system to support the addition of new protocols without being limited by protocol hierarchy. We have verified and simulated our design using the cocotb framework. The resulting architecture is implemented on Xilinx Ultrascale+ FPGAs and supports a throughput of more than 80 Gbps, with a maximum latency of only 36 ns for L4 protocol parsing. Our architecture successfully reduces latency compared to existing parsing solutions [17]. Our parser supports dynamic configuration of protocol types and extraction rules to meet the requirements of various application scenarios. In summary, our dynamic and configurable low-latency parser offers an innovative solution for programmable networks. By overcoming the fixed limitations of traditional parsers, our parser achieves high flexibility and scalability, delivering outstanding performance in high-speed network environments. This is of great significance for the parsing and processing of complex protocols and provides strong support for the development of future networks.

## Figures and Tables

**Table 1:** Resource utilization.

	Proposed DCPP	Non-Parallel [29]	[28]
LUTs	2587	2724	5945
Registers	2395	5060	5334

**Table 2:** Comparison of existing parsers.

	Datapath Width (bit)	Frequency (MHz)	Cycle Number	Latency (ns)
Our work${}_{1}$	512	250	9	36
Our work${}_{2}$	512	200	9	45
[17]	512	195.3	n.c.	46.1
[14]	512	320	30	96
[28]	512	200	n.c.	1200

## Data Availability

All the necessary data are included in the article.

## References

[1] Sha M., Guo Z., Song M. (2021). A Review of FPGA’s Application in High-speed Network Processing. J. Netw. New Media.

[2] Sivaraman A., Mason T., Panda A., Netravali R., Kondaveeti S.A. (2020). Network architecture in the age of programmability. ACM SIGCOMM Comput. Commun. Rev..

[3] Arashloo M.T., Ghobadi M., Rexford J., Walker D. HotCocoa: Hardware Congestion Control Abstractions. Proceedings of the the 16th ACM Workshop.

[4] Caulfield A., Chung E., Putnam A., Angepat H., Fowers J., Heil S., Kim J.Y., Lo D., Papamichael M., Massengill T. A cloud-scale acceleration architecture. Proceedings of the 2016 49th Annual IEEE/ACM International Symposium on Microarchitecture (MICRO).

[5] Caulfield A., Costa P., Ghobadi M. Beyond SmartNICs: Towards a fully programmable cloud. Proceedings of the 2018 IEEE 19th International Conference on High Performance Switching and Routing (HPSR).

[6] Pontarelli S., Bifulco R., Bonola M., Cascone C., Spaziani M., Bruschi V., Sanvito D., Siracusano G., Capone A., Honda M. Flowblaze: Stateful packet processing in hardware. Proceedings of the Networked Systems Design and Implementation.

[7] Bosshart P., Gibb G., Kim H.S., Varghese G., Mckeown N., Izzard M., Mujica F., Horowitz M. Forwarding Metamorphosis: Fast Programmable Match-Action Processing in Hardware for SDN. Proceedings of the Acm Sigcomm Conference on Sigcomm.

[8] Yazdinejad A., Parizi R.M., Bohlooli A., Dehghantanha A., Choo K.K.R. (2020). A high-performance framework for a network programmable packet processor using P4 and FPGA. J. Netw. Comput. Appl..

[9] Calarco G., Raffaelli C., Schembra G., Tusa G. Comparative analysis of smp click scheduling techniques. Proceedings of the International Workshop on Quality of Service in Multiservice IP Networks.

[10] Michel O., Bifulco R., Retvari G., Schmid S. (2021). The programmable data plane: Abstractions, architectures, algorithms, and applications. ACM Comput. Surv. (CSUR).

[11] Ibanez S., Brebner G., McKeown N., Zilberman N. The p4-> netfpga workflow for line-rate packet processing. Proceedings of the 2019 ACM/SIGDA International Symposium on Field-Programmable Gate Arrays.

[12] Li B., Tan K., Luo L., Peng Y., Luo R., Xu N., Xiong Y., Cheng P., Chen E. Clicknp: Highly flexible and high performance network processing with reconfigurable hardware. Proceedings of the 2016 ACM SIGCOMM Conference.

[13] Wang H., Soulé R., Dang H.T., Lee K.S., Shrivastav V., Foster N., Weatherspoon H. P4fpga: A rapid prototyping framework for p4. Proceedings of the Symposium on SDN Research.

[14] Cornevaux-Juignet F., Arzel M., Horrein P.H., Groléat T., Person C. Open-source flexible packet parser for high data rate agile network probe. Proceedings of the 2017 IEEE Conference on Communications and Network Security (CNS).

[15] Kozanitis C., Huber J., Singh S., Varghese G. Leaping multiple headers in a single bound: Wire-speed parsing using the Kangaroo system. Proceedings of the 2010 Proceedings IEEE INFOCOM.

[16] Attig M., Brebner G. 400 Gb/s programmable packet parsing on a single FPGA. Proceedings of the 2011 ACM/IEEE Seventh Symposium on Architectures for Networking and Communications Systems.

[17] Benácek P., Pu V., Kubátová H. P4-to-vhdl: Automatic generation of 100 gbps packet parsers. Proceedings of the 2016 IEEE 24th Annual International Symposium on Field-Programmable Custom Computing Machines (FCCM).

[18] Cabal J., Benáček P., Kekely L., Kekely M., Puš V., Kořenek J. Configurable FPGA packet parser for terabit networks with guaranteed wire-speed throughput. Proceedings of the 2018 ACM/SIGDA International Symposium on Field-Programmable Gate Arrays.

[19] Mashreghi-Moghadamy P., Ould-Bachirz T., Savariay Y. A Templated VHDL Architecture for Terabit/s P4-programmable FPGA-based Packet Parsing. Proceedings of the 2022 IEEE International Symposium on Circuits and Systems (ISCAS).

[20] Santiago da Silva J., Boyer F.R., Langlois J.P. P4-compatible high-level synthesis of low latency 100 Gb/s streaming packet parsers in FPGAs. Proceedings of the 2018 ACM/SIGDA International Symposium on Field-Programmable Gate Arrays.

[21] Brebner G., Jiang W. (2014). High-Speed Packet Processing using Reconfigurable Computing. IEEE Micro.

[22] Zolfaghari H., Rossi D., Nurmi J. (2020). A custom processor for protocol-independent packet parsing. Microprocess. Microsyst..

[23] Zolfaghari H., Rossi D., Nurmi J. An explicitly parallel architecture for packet parsing in software defined networks. Proceedings of the 2018 IEEE 29th International Conference on Application-specific Systems, Architectures and Processors (ASAP).

[24] Hsu K.S., Shen C.A. (2023). The design of a configurable and low-latency packet parsing system for communication networks. Telecommun. Syst..

[25] Gibb G., Varghese G., Horowitz M., McKeown N. Design principles for packet parsers. Proceedings of the Architectures for Networking and Communications Systems.

[26] Pus V., Kekely L., Korenek J. Low-latency modular packet header parser for FPGA. Proceedings of the Eighth ACM/IEEE symposium on Architectures for Networking and Communications Systems.

[27] Liu H., Qiu Z., Pan W., Li J., Huang J. HyperParser: A High-Performance Parser Architecture for Next Generation Programmable Switch and SmartNIC. Proceedings of the 5th Asia-Pacific Workshop on Networking (APNet 2021).

[28] Wang K., Guo Z., Song M., Sha M. (2022). 100 Gbps Dynamic Extensible Protocol Parser Based on an FPGA. Electronics.

[29] Li J., Han B., Sun Z., Li T., Wang X. (2019). Exploiting packet-level parallelism of packet parsing for FPGA-based switches. IEICE Trans. Commun..

[30] Zolfaghari H., Rossi D., Nurmi J. Reducing crossbar costs in the match-action pipeline. Proceedings of the 2019 IEEE 20th International Conference on High Performance Switching and Routing (HPSR).

[31] Sharif M. (2018). Programmable Data Plane at Terabit Speeds. https://conferences.sigcomm.org/sigcomm/2018/files/slides/p4/P4Barefoot.pdf.

[32] Feamster N., Rexford J., Zegura E. (2013). The Road to SDN. Queue.

[33] Liatifis A., Sarigiannidis P., Argyriou V., Lagkas T. (2023). Advancing sdn from openflow to p4: A survey. ACM Comput. Surv..

[34] Hui Y., Zhenqian F., Junnan L. (2019). Parallel Multi-Issue Programmable Parser Based on FPGA. Comput. Eng. Sci..

